# Temporal trends of incident diabetes mellitus and subsequent outcomes in patients receiving kidney transplantation: a national cohort study in Taiwan

**DOI:** 10.1186/s13098-020-00541-3

**Published:** 2020-04-28

**Authors:** Hsuan Yeh, Chihung Lin, Yan-Rong Li, Chieh-Li Yen, Cheng-Chia Lee, Jung-Sheng Chen, Kuan-Hsing Chen, Ya-Chun Tian, Pi-Hua Liu, Ching-Chung Hsiao

**Affiliations:** 1 Kidney Research Center and Department of Nephrology, Chang Gung Memorial Hospital, Linkou Branch, Taoyuan, Taiwan; 2 College of Medicine, Chang Gung University, Taoyuan, Taiwan; 3 Center for Artificial Intelligence in Medicine, Chang Gung Memorial Hospital at Linkou, Taoyuan, Taiwan; 4 Division of Endocrinology and Metabolism, Department of Internal Medicine, Linkou Chang Gung Memorial Hospital, Taoyuan, Taiwan; 5 Clinical Informatics and Medical Statistics Research Center, College of Medicine, Chang Gung University, Taoyuan, Taiwan; 6 Department of Nephrology, New Taipei Municipal TuCheng Hospital, New Taipei, Taiwan

**Keywords:** Post-transplantation diabetic mellitus, Kidney transplantation, Graft failure

## Abstract

**Background:**

Allograft kidney transplantation has become a treatment of choice for patients with end-stage renal disease (ESRD), and post-transplant diabetes mellitus (PTDM) has been associated with impaired patient and graft survival. Taiwan has the highest incidence and prevalence rates of ESRD with many recipients and candidates of kidney transplantation. However, information about the epidemiologic features of PTDM in Taiwan is incomplete. Therefore, we aimed to investigate the prevalence and incidence of PTDM with subsequent patient and graft outcomes.

**Methods:**

Using the Taiwan National Health Insurance Research Database (NHIRD), 3663 kidney recipients between 1997 and 2011 were enrolled. We calculated the cumulative incidences of diabetes mellitus (DM) after transplantation. Cox proportional hazards model with competing risk analysis was used to calculate the hazard ratio (HR) and 95% confidence intervals (CI) between three targeted groups (DM, PTDM, non-DM). The outcomes of primary interest were the occurrence of graft failure excluding death with functioning graft, all-cause mortality, death with functioning graft and major adverse cardiovascular events (MACE) including myocardial infarction (MI), cerebrovascular accident (CVA) and congestive heart failure (CHF). Subgroup analysis for graft failure excluding death with functioning graft, MACE and all-cause mortality was performed, and interaction between PTDM and recipient age was examined.

**Results:**

Of 3663 kidney transplant recipients, 531 (14%) had pre-existing DM and 631 (17%) developed PTDM. Compared with non-DM group, the PTDM and DM groups exhibited higher risk of graft failure excluding death with functioning graft (PTDM: HR 1.65, 95% CI 1.47–1.85; DM: HR 1.33, 95% CI 1.18–1.50), MACE (PTDM: HR 1.51, 95% CI 1.31–1.74; DM: HR 1.64, 95% CI 1.41–1.9), all-cause mortality (PTDM: HR 1.79, 95% CI 1.59–2.01; DM: HR 2.03, 95% CI 1.81–2.18), and death with functioning graft (PTDM: HR 1.94, 95% CI 1.71–2.20; DM: HR 1.94, 95% CI 1.71–2.21). Both PTDM and DM groups had increased cardiovascular disease-related mortality (PTDM: HR 2.14, 95% CI 1.43–3.20, p < 0.001; DM: HR 1.89, 95% CI 1.25–2.86, p = 0.002), cancer-related mortality (PTDM: HR 1.56, 95% CI 1.18–2.07, p = 0.002; DM: HR 1.89, 95% CI 1.25–2.86, p = 0.027), and infection-related mortality (PTDM: HR 1.47, 95% CI 1.14–1.90, p = 0.003; DM: HR 2.25, 95% CI 1.77–2.84, p < 0.001) compared with non-DM group. The subgroup analyses showed that the add-on risks of MACE and mortality from PTDM were mainly observed in patients who were younger and those without associated comorbidities including atrial fibrillation, cirrhosis, CHF, and MI. Age significantly modified the association between PTDM and MACE (p_interaction_ < 0.01) with higher risk in recipients with PTDM aged younger than 55 years (adjusted HR 1.64, 95% CI 1.40–1.92, p < 0.001). A trend (p_interaction_ = 0.06) of age-modifying effect on the association between PTDM and all-cause mortality was also noted with higher risk in recipients with PTDM aged younger than 55 years.

**Conclusions:**

In the present population-based study, the incidence of PTDM peaked within the first year after kidney transplantation. PTDM negatively impacted graft and patient outcomes. The magnitude of cardiovascular and survival disadvantages from PTDM were more pronounced in recipients aged less than 55 years. Further trials to improve prediction of PTDM and to prevent PTDM are warranted.

## Background

End-stage renal disease (ESRD) is a leading cause of morbidity and mortality worldwide. Taking care of patients with ESRD caused great expenditure and has become a financial burden to healthcare system [1, 2]. According to US Renal Data System (USRDS) 2018 Annual Data Report, Taiwan reported the highest incidence (493 per million population) and prevalence (3392 per million population) rate of ESRD in 2016 [3]. Allograft kidney transplantation has become a treatment of choice for patients with ESRD which is more cost-effective and has less impact on patients’ quality of life as compared with dialysis [4]. Following the introduction of cyclosporin and other novel immunosuppressive regimens, the outcomes of kidney transplant recipients have been improving. Although the patient survival after kidney transplant is superior to that of patients on maintenance dialysis, the survival of the recipients is still worse than the general population, and improving the survival of transplant patients remains an important goal of post-transplant care [5]. Post-transplantation diabetes mellitus (PTDM) was reported to correlate with poorer patient and graft survival [6, 7]. In Taiwan, there was only one single-center study of 358 kidney transplant recipients from 1986 to 2006 assessing the local incidence and risk factors of PTDM [8], but hitherto no nationwide study of the issue is available. Thus, a comprehensive understanding of the native epidemiology of PTDM and clinical observation of its role in the transplant outcomes is desirable. The National Health Insurance (NHI) program has been implemented in Taiwan since March 1995 under the Bureau of NHI of the Department of Health. The Taiwan NHI Research Database (NHIRD) contains all medical behaviors and services recorded by codes for the International Classification of Diseases, Ninth Revision (ICD-9th) and has been used widely in academic studies [9]. With the use of the NHIRD, the present study evaluated the incidence of PTDM and analyzed its associated impacts on transplant outcomes including graft failure, MACE and mortality in Taiwanese population.

## Materials and methods

### Data source

We conducted a retrospective cohort study with longitudinal data from the NHIRD. The NHI program is a nationwide, compulsory healthcare program covering approximately 99.9% of Taiwan’s population, which stood at approximately 23.37 million people in 2014. Before 2000, diagnoses in claims data were coded with A codes, followed by International Classification of Diseases, Ninth Revision, Clinical Modification (ICD-9-CM) codes thereafter. The NHIRD involves comprehensive healthcare information on insured patients, comprising disease diagnoses, inpatient orders, outpatient visits, drug prescriptions, and registries of beneficiaries with specific conditions, but it does not include laboratory data. The requirement to obtain informed consent was waived because data in the NHIRD that could identify specific patients are scrambled and encrypted before being released to researchers. However, consistent data encryption makes linking and continuously following all claims belonging to the same patient within the NHIRD feasible. The study protocol was approved by the Institutional Review Board, Chang Gung Medical Foundation, Taiwan (IRB No: 201900806B0).

In the NHI program, patients with specific chronic conditions, including organ transplantation, ESRD, malignancies, were qualified for a catastrophic illness certificate. To qualify for a certificate, a patient’s condition must be repeatedly verified by a peer review group based on clinical evidence, pathologic findings, and laboratory data. The Registry for Catastrophic Illness Patient Database (RCIPD) is an NHIRD subset composed of certificated patients’ data, including those receiving kidney transplantation or maintenance dialysis.

### Patient selection and study design

**Fig. 1:**
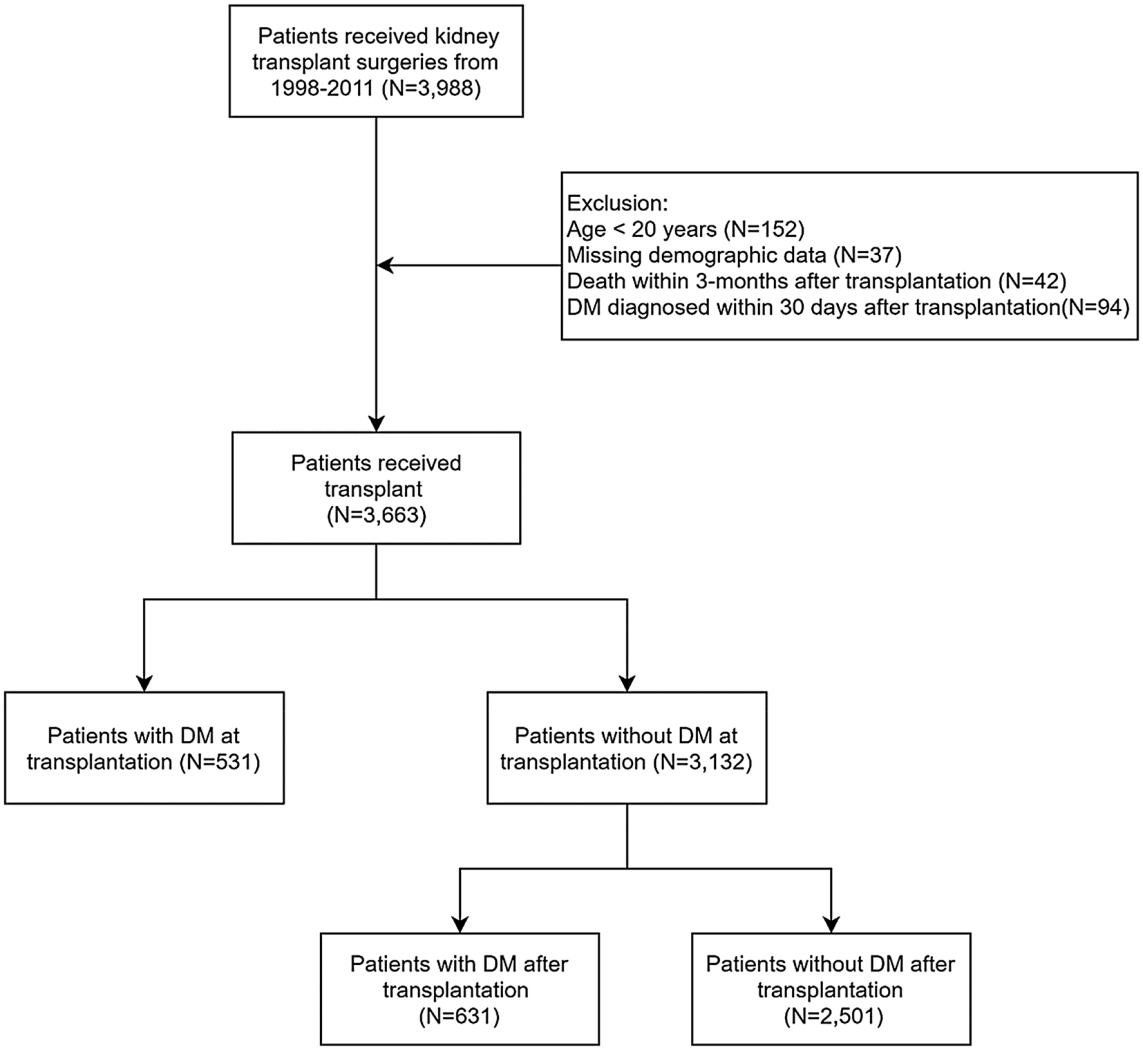
The flow chart of patient selection process. *DM* diabetes mellitus

Figure 1 demonstrates the process used for selecting the participants in the study cohorts, and Additional file 1: Figure S1 shows our study design. Patients who received kidney transplantation (ICD-9-CMcode = V420, 996.81; surgical code = 76020A, 76020B, 97416K, 97417A, 97418B) from January 1, 1998 to December 31, 2011 were identified from the Taiwan NHIRD. Index date was defined as the date of kidney transplantation. The diagnosis of diabetes mellitus (DM) was defined by at least two outpatient claims or one inpatient claim with ICD-9-CM code = 250 and use of at least one of the oral anti-diabetic agents including metformin, sulfonylurea, glinides, thiazolidinediones (TZD), acarbose, dipeptidyl peptidase-4 inhibitors (DPP4-inhibitors) or insulin. We excluded patients with age < 20 years (N = 156), or missing demographic data (N = 37). Because surgical complications or infections related to high dose immunosuppressants caused some patients deaths within several months after transplantation, patients who died within 3 months after transplantation were excluded (N = 42). Patients newly diagnosed with DM within 30 days after transplantation were also excluded to improve the diagnostic accuracy and to eliminate the transient hyperglycemia induced by high-dose glucocorticoids and surgical stress (N = 94). Finally, 3663 patients received kidney transplant were enrolled in the study cohort. Among 3663 patients, 531 (14%) patients were diagnosed with DM before transplant and 631 (17%) patients were diagnosed with PTDM.

### Covariates and study outcomes

Diseases were detected using A codes before 2000 or ICD-9-CM diagnostic codes after 2000. The covariates were age, sex, place of residence, income levels, occupations, comorbidities, Charlson comorbidity index (CCI), medications, rejection, cytomegalovirus (CMV) infection and initial dialysis type.

Comorbidities were identified when reported for more than two outpatient visits or one inpatient stay within the previous year of index date. Comorbidities included malignancy, hypertension, hyperlipidemia, cerebrovascular accident (CVA), myocardial infarction (MI), CHF, atrial fibrillation (AF), peripheral artery disease, chronic obstructive pulmonary disease (COPD), liver cirrhosis, hepatitis B (HBV) infection and hepatitis C (HCV) infection. Most diagnostic codes used for these comorbidities had been validated in previous NHIRD-based studies [10, 11]. Medications were recognized by the filling of a prescription at least twice or refilling a prescription for a chronic illness at least once after the index date. Medications included steroid, cyclosporin, tacrolimus, mycophenolate mofetil or mammalian target of rapamycin inhibitor (m-TOR inhibitor) like everolimus or sirolimus. Some peritoneal dialysis patients temporarily underwent hemodialysis at the initiation of dialysis; therefore, dialysis modality was defined as the modality at day 90 after the first dialysis session.

The outcomes of primary interest were all-cause mortality, death with functioning graft, the occurrence of graft failure excluding death with functioning graft, and MACE including MI, CHF and CVA. Graft failure was defined as returning to hemodialysis or peritoneal dialysis after kidney transplant for more than 90 days. Components of MACE was detected based on the principal diagnosis of an emergency visit or hospitalization. Mortality was defined as withdrawal from the NHI program. Given that the association between DM status and mortality may be modified by events that occur after graft loss, death with functioning graft was also included as a primary endpoint, defined as the mortality group with elimination of those who had been on maintenance hemodialysis continuously for more than 3 months before death. The secondary outcomes were cardiovascular diseases-related, infection-related and cancer-related death. These outcomes were detected based on the principal or secondary diagnosis of an emergency department visit or hospitalization. Components of cardiovascular diseases were detected based on the principal diagnosis of any emergency visit or hospitalization, most of these diagnostic codes for which have been previously validated [12, 13]. All participants were followed from the index date until the first occurrence of graft failure, MACE, death, or the end of the follow-up period (December 31, 2013), whichever came first.

### Statistical analysis

The descriptive statistics of demographic and clinical information at the date of transplantation were stratified by non-DM, PTDM and DM groups. The difference between groups was estimated by Chi square test for categorical variables and one-way analysis of variance (ANOVA) for continuous variables. The temporal trends of incidence rates of DM prior to and after kidney transplantation were calculated by each year. The Cox proportional hazards model was used to estimate the risk of incident DM after transplantation using demographic and clinical variables in the patients without history of DM while adjusting for the covariates. Since some studies revealed that the major impact of diabetes status on graft survival was not due to rejection but to death instead [14], it is crucial to highlight the interested graft outcome in this study was the graft survival unaffected by death of the individuals, i.e., graft failure excluding death with functioning graft. As a result, we performed competing risk analysis using sub-distributional hazards model to estimate the outcome of graft failure excluding death with functioning graft. Similarly, for the outcome of MACE, we performed competing risk analysis with those who deceased without experiencing MACE events censored in an informative manner.

The incident DM status after transplantation was time-dependent rather than a fixed categorical variable, which violated the assumption of Cox proportional model. Therefore, the Simon and Makuch method and Mantel-Byar test, rather than the traditional Kaplan–Meier method and log-rank test, were used to draw survival curves for the non-DM, PTDM and DM groups. To clarify the association between the occurrence of PTDM and outcomes, we stratified the risks of primary interests by era. Risks of graft failure excluding death with functioning graft, MACE and overall mortality in 0–3 years and > 3 years after transplantation were calculated. From the time-dependent Cox model with consideration of the competing risk of death and inverse probability of treatment weighting (IPW) using the propensity score, the adjusted hazard ratios (aHRs) with 95% confidence intervals (CIs) were calculated for the associations between DM groups and interested outcomes.The propensity scores used for IPW were estimated with generalized boosted regression models and considered the demographics, CCI, comorbidities prior and after transplantation, plus medications [15]. The subgroup analysis of Cox models was conducted for each stratified groups of age, sex, CCI and significant comorbidities. All statistical analyses were performed using R language and SAS 9.4 software (SAS Institute Inc., Cary, NC, USA). A statistical significance was considered for two-sided p-value < 0.05 for all tests.

## Results

### Subject characteristics

Table 1 presents the baseline characteristics of the DM group when compared with the PTDM group and non-DM group. Overall, a total of 3663 kidney transplant patients met the inclusion criteria and were categorized by their DM status into the DM (N = 531), PTDM (N = 631), or non-DM (N = 2501) groups. Type 1 DM only existed in the pre-existing DM group, with 33 out of 531 recipients. The mean age (years) was 52.19 ± 9.5 in the DM group, 50.62 ± 10.2 in the PTDM group and 43.15 ± 10.94 in the non-DM group, respectively (p < 0.001). In general, the DM group was the oldest, had the highest proportion of cardiovascular comorbidities including hypertension, hyperlipidemia, MI, CVA, peripheral artery disease, and carried the highest CCI, followed by the PTDM group, and then the non-DM group.

**Table 1:** Demographical characteristic in patients with kidney transplantation by diabetes status

Characteristics	DM status						p-value
	DM		PTDM		Non-DM		
No. of patients	531		631		2501		
Male, N (%)	379	(71.4)	339	(53.7)	1195	(47.8)	< 0.001
Age years, mean ± SD	52.19 ± 9.50		50.62 ± 10.12		43.15 ± 10.94		< 0.001
Observation years, mean ± SD	6.21 ± 3.37		5.19 ± 3.60		7.77 ± 3.90		< 0.001
CCI scores^a^, N (%)							
Mean ± SD	0.81 ± 1.00		0.83 ± 1.41		0.54 ± 0.87		< 0.001
0	261	(49.2)	341	(54.0)	1559	(62.3)	< 0.001
1	154	(29.0)	171	(27.1)	670	(26.8)	
2+	116	(21.9)	119	(18.9)	272	(10.9)	
Place of residence, N (%)							
Urban	340	(64.0)	380	(60.2)	1671	(66.8)	0.032
Suburban	156	(29.4)	210	(33.3)	680	(27.2)	
Rural	35	(6.6)	41	(6.5)	150	(6.0)	
Income levels, N (%)							
Quintile 1	99	(18.6)	126	(20.0)	439	(17.6)	< 0.001
Quintile 2	95	(17.9)	187	(29.6)	685	(27.4)	
Quintile 3	99	(18.6)	61	(9.7)	396	(15.8)	
Quintile 4	102	(19.2)	127	(20.1)	505	(20.2)	
Quintile 5	136	(25.6)	130	(20.6)	476	(19.0)	
Occupation^a^, N (%)							
1	89	(16.8)	83	(13.2)	401	(16.0)	0.073
2	28	(5.3)	36	(5.7)	145	(5.8)	
3	140	(26.4)	208	(33.0)	814	(32.6)	
4	213	(40.1)	249	(39.5)	917	(36.7)	
5	61	(11.5)	55	(8.7)	224	(9.0)	
Comorbidities, N (%)							
Malignancy	15	(2.8)	59	(9.4)	67	(2.7)	< 0.001
Hypertension	379	(71.4)	371	(58.8)	1290	(51.6)	< 0.001
Hyperlipidemia	211	(39.7)	164	(26.0)	387	(15.5)	< 0.001
Stroke	54	(10.2)	25	(4.0)	75	(3.0)	< 0.001
Myocardial infarction	11	(2.1)	4	(0.6)	8	(0.3)	<.001
Congestive heart failure	105	(19.8)	60	(9.5)	250	(10.0)	< 0.001
Peripheral vascular disease	41	(7.7)	31	(4.9)	81	(3.2)	< 0.001
Atrial fibrillation	11	(2.1)	6	(1.0)	15	(0.6)	0.004
COPD	33	(6.2)	52	(8.2)	125	(5.0)	0.007
Liver cirrhosis	14	(2.6)	30	(4.8)	42	(1.7)	< 0.001
HBV	4	(0.8)	3	(0.5)	6	(0.2)	0.167
HCV	22	(4.1)	6	(1.0)	4	(0.2)	< 0.001
Medication, N (%)							
Cyclosporin	261	(49.2)	185	(29.3)	1111	(44.4)	< 0.001
Tacrolimus	378	(71.2)	436	(69.1)	1921	(76.8)	< 0.001
Mycophenolate	366	(68.9)	385	(61.0)	1766	(70.6)	< 0.001
m-TOR inhibitor	230	(43.3)	296	(46.9)	1265	(50.6)	0.005
Steroid							
Non-use	4	(0.8)	79	(12.5)	4	(0.2)	< 0.001
≤ 10 mg/day	408	(76.8)	426	(67.5)	2115	(84.6)	
> 10 mg/day	119	(22.4)	126	(20.0)	382	(15.3)	
Comorbidities after transplantation, N (%)							
Rejection	317	(59.7)	294	(46.6)	1345	(53.8)	< 0.001
CMV infection	55	(10.4)	37	(5.9)	212	(8.5)	0.019
Initial dialysis type, N (%)							
Hemodialysis	423	(79.7)	481	(76.2)	1656	(66.2)	< 0.001
Peritoneal dialysis	24	(4.5)	39	(6.2)	212	(8.5)	
Both	77	(14.5)	104	(16.5)	606	(24.2)	

*No. or N* number, *DM* diabetic mellitus, *PTDM* post-transplantation diabetic mellitus, *SD* standard deviation, *CCI* Charlson comorbidity index, *COPD* chronic obstructive pulmonary disease, *HBV* hepatitis B virus, *HCV* hepatitis C virus, *m-TOR* mammalian target of rapamycin, CMV cytomegalovirusa, *CCI* Charlson comorbidity index, except diabetic mellitus and renal disease)

^a^Occupation categories: 1. dependents; 2. civil servants, teachers, military personnel and veterans; 3. non-manual workers and professionals; 4. manual workers and 5. other

### Temporal trends of incident DM rates in patients before and after kidney transplantation

**Fig. 2:**
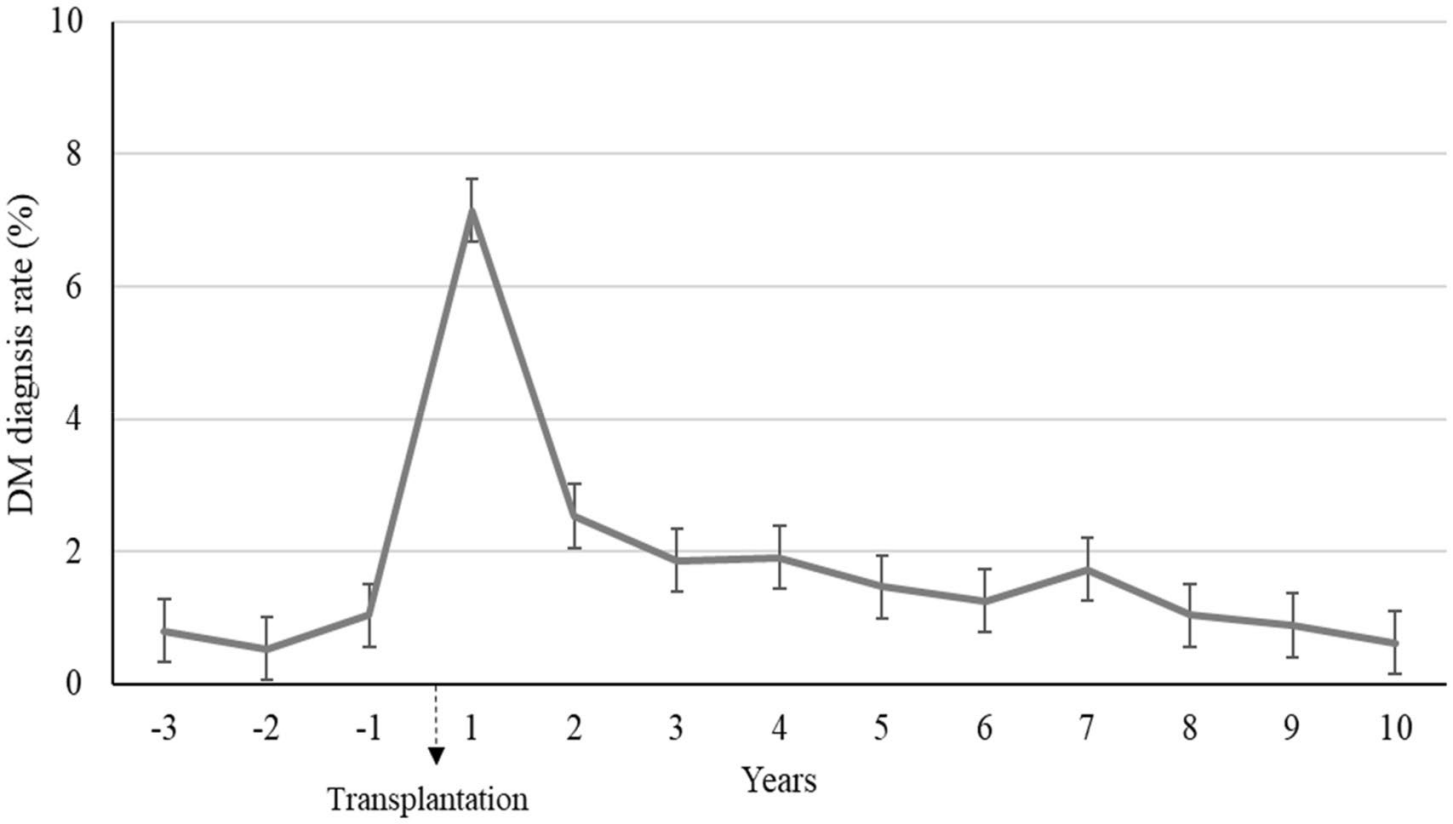
Temporal trends of DM rates prior and after transplantation. *DM* diabetes mellitus, *PTDM* post-transplant diabetes mellitus, *IQR*, interquartile range. *The cumulative incidences of DM after transplantation (or PTDM) were 7.2% for 1-year, 11.5% for 3-year, 15% years for 5-year, and 23.4% for 10-year. The median (IQR) capture time of PTDM development was 2.4 (0.4–5.8) years

Figure 2 shows the temporal trends of incidence of DM in patients before and after kidney transplantation. Before kidney transplantation, the incidence of DM was around 1% per year. The incidence of DM was the highest in the first year after kidney transplantation, reaching 7.2%. The median time to the development of PTDM was 2.4 (0.4–5.8) years. Overall, the incidence of DM was higher in the post-transplantation period than in the pre-transplantation period. The cumulative incidences of DM after transplantation were 7.2% for 1-year, 11.5% for three-year, 15% years for 5-year, and 23.4% for 10-year.

### Risk factors of PTDM among 3132 patients without DM prior to transplantation

The risk factors of PTDM among 3132 patients without DM prior to transplantation were shown in Additional file 1: Table S1. The Cox proportional hazards model with competing risk analysis revealed advanced age (aHR = 1.03; 1.03–1.04; p < 0.001) and male sex (aHR = 1.20; 1.03–1.43; p = 0.02) were associated with increased risk of PTDM. Hyperlipidemia (aHR = 1.43; 1.15–1.75; p < 0.001) and malignancy (aHR = 1.65; 1–2.72; p = 0.048) predisposed patients to higher risks of PTDM.

### Risk of graft failure, MACE, and all-cause mortality for non-DM, PTDM and DM groups

**Fig. 3:**
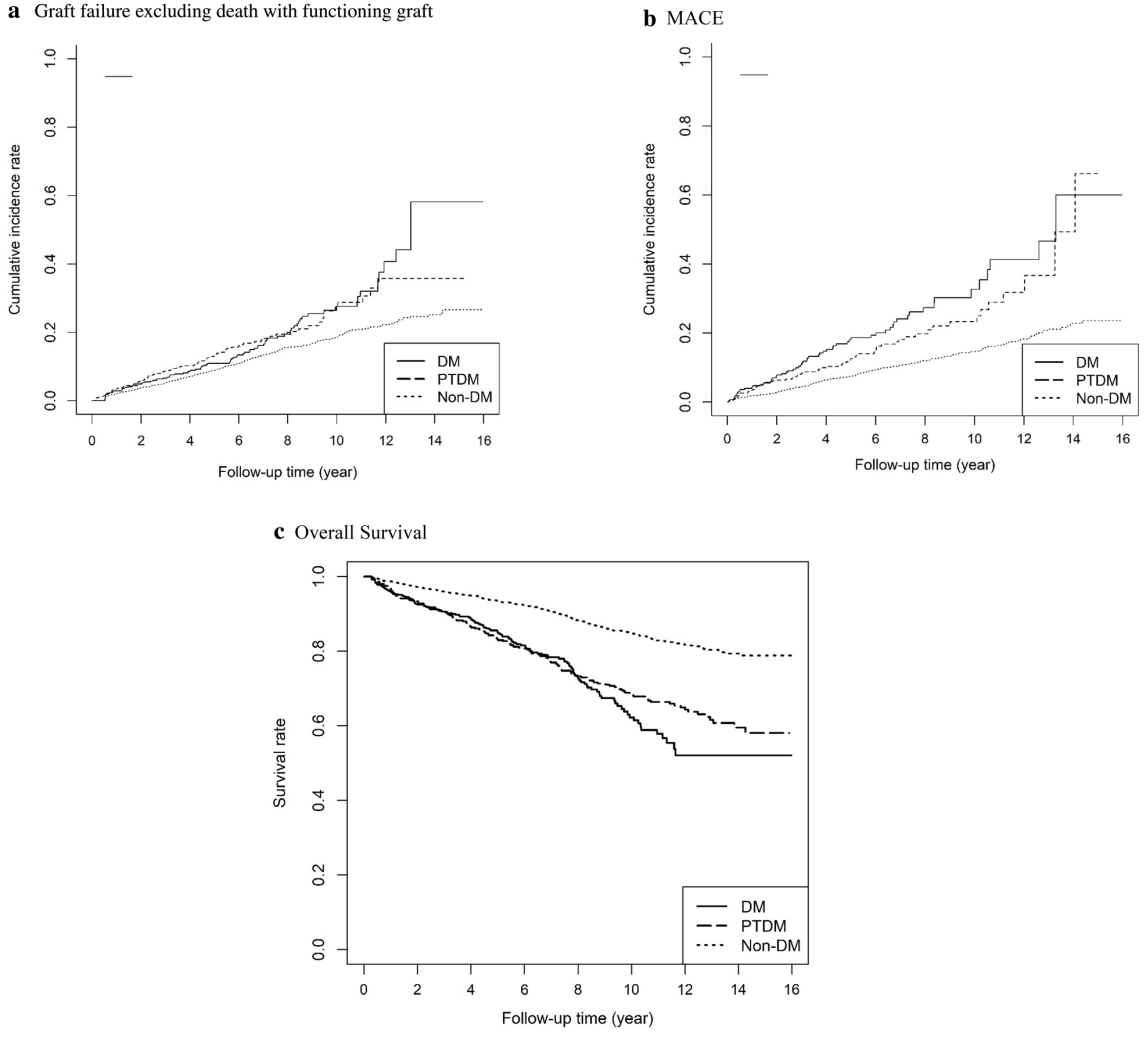
Cumulative incidence rates of graft failure excluding death with functioning graft, MACE and survival curves in DM, PTDM, and non-DM groups (all p-values < 0.05 for Mantel-Byar test). **a** Graft failure excluding death with functioning graft. **b** MACE. **c** Overall survival. DM diabetes mellitus, *PTDM* post-transplant diabetes mellitus, *MACE*, major adverse cardiovascular events

Table 2 presents the risk of graft failure, MACE, all-Cause mortality and death with functioning graft in three targeted groups (non-DM, PTDM, DM). IPW-standardized time-dependent Cox proportional hazards model with competing risk analysis was used to calculate the HR and 95% CIs between three targeted groups. Regarding graft failure excluding death with functioning graft, the PTDM group and the DM group exhibited higher risks compared with the non-DM group (PTDM: HR 1.65, 95% CI 1.47–1.85; DM: HR 1.33, 95% CI 1.18–1.50). Regarding MACE, the PTDM group and the DM group had higher risks compared with the non-DM group (PTDM: HR 1.51, 95% CI 1.31–1.74; DM: HR 1.64, 95% CI 1.41–1.90). Regarding all-cause mortality, the PTDM group and the DM group had higher risks compared with the non-DM group (PTDM:HR 1.79, 95% CI 1.59–2.01; DM: HR 2.03, 95% CI 1.81–2.18). Regarding death with functioning graft, the PTDM group and the DM group demonstrated higher risks compared with the non-DM group (PTDM: HR 1.94, 95% CI 1.71–2.20; DM: HR 1.94, 95% CI 1.71–2.21). The cumulative survival curves for graft survival, MACE and all-cause mortality using the Simon-Makuch method are shown in Fig. 3. Risks of graft failure excluding death with functioning graft, MACE, and all-cause mortality in the targeted groups stratified by time period after transplantation were shown in the Additional file 1: Table S2.

**Table 2:** Risk of graft failure, MACE, all-cause mortality, and death with functioning graft in kidney transplant recipients stratified by diabetes status

DM Status^a^	Graft failure				MACE				All-cause mortality		Death with functioning graft	
	Cox model		Cox model and competing risk		Cox model		Cox model and competing risk		Cox model		Cox model	
	HR (95% CI)	P	HR (95% CI)	p	HR (95% CI)	p	HR (95% CI)	p	HR (95% CI)	p	HR (95% CI)	p
Non-DM	(Reference)		(Reference)		(Reference)		(Reference)		(Reference)		(Reference)	
PTDM	1.75 (1.56–1.96)	< 0.001	1.65 (1.47–1.85)	< 0.001	1.59 (1.38–1.84)	< 0.001	1.51 (1.31–1.74)	< 0.001	1.79 (1.59–2.01)	< 0.001	1.94 (1.71–2.20)	< 0.001
DM	1.40 (1.24–1.57)	< 0.001	1.33 (1.18–1.50)	< 0.001	1.74 (1.50–2.02)	< 0.001	1.64 (1.41–1.90)	< 0.001	2.03 (1.81–2.28)	< 0.001	1.94 (1.71–2.21)	< 0.001

*MACE* major adverse cardiovascular event, *HR* hazard ratio, *CI* confidence interval, *DM* diabetes mellitus, *PTDM* post-transplant diabetes mellitus

^a^All HRs (95% CI) were calculated by using Cox proportional hazards model with counting process accounted for time-dependent variables and weighted by the propensity scores. Propensity scores for the three groups stratified by DM status were calculated from variables of gender, age, Charlson comorbidity scores, place of residence, income levels, occupations, presence of comorbidities (including malignancy, hypertension, hyperlipidemia, cerebrovascular disease, myocardial infarction, congestive heart failure, peripheral vascular disease, atrial fibrillation, chronic obstructive pulmonary disease, cirrhosis, hepatitis B virus, hepatitis C virus), cyclosporin, tacrolimus, mycophenolate mofetil, mammalian target of rapamycin inhibitor, steroid, kidney transplantation rejection and cytomegalovirus infection. Competing risk of death with functioning graft was calculated as informative death-censoring mechanism for evaluating the outcome model of graft failure excluding death with functioning graft. Competing risk of death without experiencing MACE was calculated as informative death-censoring mechanism for evaluating the outcome model of MACE

### Mortality risks from cardiovascular diseases, cancer and infection for non-DM, PTDM and DM groups

Table 3 shows HRs for cardiovascular disease, cancer and infection-related death for the three targeted groups. Compared with non-DM group, both PTDM and DM groups had increased cardiovascular disease-related mortality (PTDM: HR 2.14, 95% CI 1.43–3.20, p < 0.001; DM: HR 1.89, 95% CI 1.25–2.86, p = 0.002), cancer-related mortality (PTDM: HR 1.56, 95% CI 1.18–2.07, p = 0.002; DM: HR 1.89, 95% CI 1.25–2.86, p = 0.027), and infection-related mortality (PTDM: HR 1.47, 95% CI 1.14–1.90, p = 0.003; DM: HR 2.25, 95% CI 1.77–2.84, p < 0.001).

**Table 3:** Hazard ratios for cancer, cardiovascular diseases, and infection-related death in kidney transplant recipients stratified by diabetes status

	Non-DM	PTDM	DM
Total no. of patients	2501	631	531
No. of death	336	123	134
Cancer-related death^a^			
N (%)	63 (2.5%)	25 (4.0%)	14 (2.6%)
HR (95% CI)	(REF)	1.56 (1.18–2.07)	1.39 (1.04–1.85)
p-value		0.002	0.027
CVD-related death^a^			
N (%)	25 (1.0%)	10 (1.6%)	10 (1.9%)
HR (95% CI)	(REF)	2.14 (1.43–3.20)	1.89 (1.25–2.86)
p-value		< 0.001	0.002
Infection-related death^a^			
N (%)	74 (3.0%)	28 (4.4%)	39 (7.3%)
HR (95% CI)	(REF)	1.47 (1.14–1.90)	2.25 (1.77–2.84)
p-value		0.003	< 0.001

*No. or N* number, *HR* hazard ratio, *CI* confidence interval, *DM* diabetes mellitus, *PTDM* post-transplant diabetes mellitus, *CVD* cardiovascular disease

^a^All HRs (95% CI) were calculated using Cox proportional hazards model with counting process accounted for time-dependent variables and weighted by the propensity scores

### Subgroup analysis

To further verify whether the effect of PTDM was consistent among different clinical situations, we performed pre-specified subgroup analyses for graft failure excluding death with functioning graft, MACE and all-cause mortality, as shown in Table 4. The rates of graft failure excluding death with functioning graft were higher in recipients with PTDM compared with non-DM group, especially for those aged younger than 55 years, those with low CCI (= 0) and those without comorbidities including AF, liver cirrhosis, CHF, and MI. The rates of MACE were higher in recipients with PTDM compared with non-DM group, particularly for those aged younger than 55 years, those with low CCI (≤ 1), and those without comorbidities including AF, liver cirrhosis, CHF, MI. The rates of all-cause mortality were higher in recipients with PTDM compared with non-DM group, specifically for those without comorbidities including AF, liver cirrhosis, CHF, and MI, but were consistently higher than non-DM group independent of age or CCI. To confirm the modification effect of age on the association between PTDM and outcomes of interest, we examined the interaction between PTDM status and age. The p-values for interaction (p_interaction_) between PTDM status and age were 0.463 for graft failure excluding death with functioning graft, 0.01 for MACE, and 0.06 for all-cause mortality.

**Table 4:** Subgroup analyses for the risk of interested outcomes

Category^a^	Subgroup	Outcome											
		Graft failure excluding death with functioning graft				MACE				All-cause mortality			
		PTDM		DM		PTDM		DM		PTDM		DM	
		HR (95% CI)	p	HR (95% CI)	p	HR (95% CI)	p	HR (95% CI)	p	HR (95% CI)	p	HR (95% CI)	p
Age	< 55	1.78 (1.57–2.00)	< 0.001	1.38 (1.21–1.56)	< 0.001	1.64 (1.40–1.92)	< 0.001	1.63 (1.37–1.94)	< 0.001	1.76 (1.52–2.03)	< 0.001	2.12 (1.83–2.44)	< 0.001
	> = 55	1.00 (0.71–1.40)	0.998	1.29 (0.95–1.75)	0.109	1.05 (0.78–1.41)	0.757	1.42 (1.06–1.89)	0.019	1.80 (1.47–2.19)	< 0.001	1.62 (1.33–1.97)	< 0.001
Gender	Female	1.56 (1.32–1.85)	< 0.001	1.17 (0.98–1.40)	0.089	1.61 (1.31–1.97)	< 0.001	1.69 (1.35–2.11)	< 0.001	2.05 (1.71–2.46)	< 0.001	2.45 (2.05–2.93)	< 0.001
	Male	1.69 (1.45–1.98)	< 0.001	1.44 (1.23–1.69)	< 0.001	1.40 (1.16–1.70)	0.001	1.53 (1.25–1.87)	< 0.001	1.62 (1.39–1.89)	< 0.001	1.75 (1.50–2.03)	< 0.001
CCI	0	1.94 (1.68–2.26)	< 0.001	1.11 (0.94–1.31)	0.232	1.45 (1.22–1.74)	< 0.001	1.75 (1.46–2.10)	< 0.001	1.43 (1.21–1.69)	< 0.001	2.31 (1.98–2.70)	< 0.001
	1	1.25 (1.00–1.55)	0.048	1.60 (1.30–1.96)	< 0.001	1.67 (1.29–2.17)	< 0.001	1.34 (1.00–1.81)	0.054	2.28 (1.84–2.81)	< 0.001	1.61 (1.29–2.02)	< 0.001
	2+	1.30 (0.93–1.83)	0.131	1.65 (1.21–2.26)	0.002	1.22 (0.74–2.01)	0.433	1.57 (0.93–2.63)	0.091	2.07 (1.58–2.71)	< 0.001	1.84 (1.41–2.40)	< 0.001
AF	No	1.64 (1.46–1.84)	< 0.001	1.31 (1.17–1.48)	< 0.001	1.54 (1.33–1.77)	< 0.001	1.64 (1.41–1.91)	< 0.001	1.81 (1.61–2.04)	< 0.001	2.05 (1.82–2.30)	< 0.001
	Yes	0.98 (0.51–1.89)	0.956	N/A	N/A	N/A	N/A	0.93 (0.36–2.42)	0.882	1.33 (0.43–4.13)	0.619	0.95 (0.43–2.12)	0.909
Liver cirrhosis	No	1.65 (1.47–1.86)	< 0.001	1.30 (1.15–1.47)	< 0.001	1.49 (1.30–1.72)	< 0.001	1.67 (1.44–1.95)	< 0.001	1.80 (1.59–2.02)	< 0.001	2.09 (1.86–2.35)	< 0.001
	Yes	1.38 (0.71–2.69)	0.344	2.13 (1.13–4.01)	0.019	1.93 (0.80–4.67)	0.147	0.80 (0.28–2.33)	0.683	1.47 (0.77–2.79)	0.241	0.53 (0.22–1.25)	0.146
CHF	No	1.70 (1.50–1.92)	< 0.001	1.25 (1.10–1.43)	0.001	1.51 (1.31–1.74)	< 0.001	1.64 (1.41–1.90)	< 0.001	1.86 (1.64–2.10)	< 0.001	2.12 (1.88–2.40)	< 0.001
	Yes	1.25 (0.89–1.76)	0.204	1.72 (1.26–2.34)	0.001	N/A	N/A	N/A	N/A	1.29 (0.90–1.84)	0.170	1.45 (1.05–2.00)	0.024
MI	No	1.65 (1.47–1.85)	< 0.001	1.29 (1.15–1.46)	< 0.001	1.51 (1.31–1.74)	< 0.001	1.64 (1.41–1.90)	< 0.001	1.80 (1.60–2.03)	< 0.001	2.03 (1.81–2.28)	< 0.001
	Yes	1.37 (0.71–2.66)	0.348	N/A	N/A	N/A	N/A	N/A	N/A	N/A	N/A	2.43 (0.44–13.54)	0.311
CVA	No	1.65 (1.47–1.86)	< 0.001	1.30 (1.15–1.47)	< 0.001	1.51 (1.31–1.74)	< 0.001	1.64 (1.41–1.90)	< 0.001	1.74 (1.54–1.96)	< 0.001	2.03 (1.80–2.28)	< 0.001
	Yes	0.84 (0.33–2.17)	0.720	2.49 (1.32–4.68)	0.005	N/A	N/A	N/A	N/A	3.30 (1.98–5.51)	< 0.001	1.96 (1.18–3.26)	0.009

*MACE* major adverse cardiovascular events, *DM* diabetes mellitus PTDM, post-transplant diabetes mellitus *HR* hazard ratio, *CI* confidence interval, *CCI* Charlson comorbidity index with exception of diabetes mellitus and renal disease, *AF* atrial fibrillation, *CHF* congestive heart failure, MI myocardial infarction, CVA cerebrovascular accident

^a^All HRs (95% CI) were measured and compared to the non-DM group using Cox proportional hazards model with counting process accounted for time-dependent variables and weighted by the propensity scores

## Discussion

In the present study, we conducted a retrospective cohort analysis with longitudinal data from the NHIRD. The key findings of the present study were as follows: (i) PTDM incidence peaked in the first year post-transplant; (ii) the presence of DM (pre-existing or incident) was associated with higher risks of all-cause mortality, MACE and graft failure excluding death with functioning graft; (iii) the adverse effects of PTDM on MACE and patient survival were observed exclusively in those who were relatively young and with less comorbidities.

PTDM, also known as new-onset diabetes after transplantation (NODAT), is a common complication of kidney transplantation. Due to the lack of standards regarding the definition of diagnosis, it was once difficult to estimate the incidence rate of PTDM precisely. A review of literature reported the incidence of PTDM ranged widely between 10 and 74% [16]. In 2003, the first international consensus guidelines for NODAT were published [17] based on American Diabetes Association (ADA) and World Health Organization (WHO) criteria for DM. The guidelines were later revised in 2014 with the addition of hemoglobin A1C as a criterion [18]. A large cohort from United Network for Organ Sharing/Organ Procurement and Transplantation Network (UNOS/OPTN) database found the cumulative incidence of NODAT was 9.7% 1 year after transplantation by using the consensus in 2003 as disease definition [14]. However, more recent studies showed the incidence of NODAT could be much higher with the application of revised guideline in 2014. The incidence reported in our study was more in accordance with the study from UNOS/OPTN database but lower than some recent studies. Such difference can be explained in part by the fact that some of the patients analyzed in our study were transplanted and diagnosed with DM in the era before the international consensus guidelines issued in 2003. Besides, the diagnosis of DM was defined in a relatively strict way in the present study. The incidence of PTDM in our study peaked in the first year post-transplant, and this trend was consistent with the results of an earlier study [14]. In addition, the incidence declined to 2% from the second to seventh year after transplantation but was still higher than pre-transplant status (1%). The possible explanations of higher DM occurrence include the use of immunosuppressive agents as well as the insulin resistance caused by inflammation and infection after transplantation [16, 19]. Our finding emphasizes the importance of DM screening and measures to prevent its occurrence in this period.

Since pre-transplant DM has been associated with inferior transplant outcomes [20], there is concern that PTDM may also affect outcomes after transplantation. Boudreaux et al. in 1987 was one of the first to suggest that PTDM impacts patient survival [21]. From then on, there has been a considerable amount of literature regarding the issue. PTDM was associated with worse patient survival independent of other clinical factors [6, 22–25]. In our study, patients with PTDM had higher risks of MACE and all-cause mortality. The increased mortality rate could be attributed to cardiovascular diseases, which was in good agreement with previous findings in the literature [25, 26]. In a retrospective study analyzing a large cohort(N = 1146) of first-time kidney transplant recipients followed up for 24 years, PTDM was found to be a strong independent risk factor for death, mainly from cardiovascular causes, regardless of the presence of cardiovascular diseases diagnosed before transplantation [25]. In addition to cardiovascular disease, our study revealed that the increased mortality risk in PTDM group, compared with non-DM group, was also ascribed to excess risk of infection-related death. This is not surprising since both DM and PTDM have been shown to be associated with greater risk of sepsis and sepsis-related mortality [27, 28]. Finally, our study exclusively showed that PTDM group had excess risk of cancer-related death compared with non-DM group. However, previous studies did not support this finding [27, 29]. Although DM has been widely known to increase the risk of malignancy with the postulated mechanisms including stimulation of insulin-like growth factor (IGF)-axis and increased cytokines production [30], whether this can be extrapolated to the case of PTDM remains to be elucidated.

With respect to the negative influence of PTDM on graft function, our study showed that the PTDM group exhibited a significantly higher risk of graft failure excluding death with functioning graft compared with the non-DM group even after competing risk analysis serving as death-censoring investigation. The study published by Miles et al. showed PTDM was associated with impaired long-term renal allograft function and survival, though it did not provide the information about death-censoring analysis [31]. Another research correlated favorably with our study, indicating PTDM as an independent risk factor for death-censored renal graft failure [6]. Nevertheless, a former study showed that PTDM was related to overall graft failure but not to death-censored graft failure, which was mainly attributed to acute rejection during the first year [14]. This discrepancy might be owing to not only the definition of PTDM but also the ways of detection of graft failure.

To verify whether the deleterious effects of PTDM on patient and graft survival were consistent among patients with different underlying clinical conditions, we performed subgroup analyses for graft failure excluding death with functioning graft, MACE and all-cause mortality. In patients under the age of 55 years, PTDM correlated with a significantly higher risk of MACE and a trend of higher mortality risk, while in those over the age of 55 years with PTDM had shown neither difference in MACE nor death. Our results shared similarities with some earlier studies: A retrospective study in the United Kingdom revealed that patient survival was adversely affected by both pre-existing diabetes and by PTDM, particularly in those with an age less than 55 years. However, the cause of death was not identified in the study [22]. Similarly, a population cohort study of kidney transplant recipients recruited in Australia and New Zealand Dialysis and Transplant Registry found that HRs for all-cause mortality and death with functioning graft in recipients carrying DM were significantly higher than non-DM group. The highest risk was shown in recipients with DM aged younger than 40 years especially. Risk was increased to a lesser extent in recipients with DM older than 55 years old [27]. Although this study did not differentiate the pre-existing DM and PTDM, it provided some evidence that the magnitude of the excess risk associated with DM status was more pronounced in young group. Consequently, we can infer from the studies listed above that there is probably some “age-group-specific” phenomenon for deleterious effects of PTDM regarding patient survival or life-threatening complications. Interestingly, our study also discovered that PTDM was universally associated with higher risks of death, graft failure excluding death with functioning graft and MACE in patients without AF, cirrhosis, CHF, MI, and CVA. However, PTDM did not further increase the risks in patients with the listed comorbidities, except for the CVA subgroup, in which PTDM consistently attributed to a higher risk of death irrelevant to the existence of CVA. Taken together, our findings suggested that those with longer life expectancy and better baseline physical condition are more susceptible to the negative impacts of PTDM.

Concerning the detrimental effect of PTDM on patient survival and possibly graft survival, there have been plenty of studies evaluating the potential measures to prevent PTDM, including lifestyle modification [32–34], steroid avoidance [35–38] or calcineurin-inhibitor-sparing regimen [39, 40]. Moreover, there are ongoing clinical trials assessing the efficacy of early management of hyperglycemia with basal insulin (NCT01683331) and sitagliptin (NCT01928199) for the prevention of PTDM. A double-blinded randomized controlled trial is currently conducted to value the effect of vitamin D3 (cholecalciferol) supplementation on the incidence of PTDM (NCT01431430), and the results are still pending. With emerging groups working on the issue and a great hope for better strategies to prevent PTDM, clinicians might also need to take the rising healthcare cost into account. Our subgroup analysis revealed that young people and those with less comorbidities tended to be more affected by PTDM. Although the reason of such trend is still uncertain, our findings may help better define the candidates who are prone to benefit from a more stringent post-transplant glycemic control and even the preventive treatment of PTDM.

Admittedly, our research had some limitations. First, our study was subject to flaws inherent in retrospective analysis, such as the reporting bias and erroneous data. Second, because NHIRD does not provide information about all patient characteristics related to our primary interest, certain well-established risk factors including donor factors and human leukocyte antigen (HLA) matching were inevitably veiled. Third, because NHIRD does not include laboratory data, our study was limited by lack of information needed to completely meet the diagnostic criteria of the consensus guidelines. The alternative solution was to filter all the diagnoses made and recorded by clinicians and to examine whether they matched the logic behind the consensus recommendations. In the present study, the diagnosis of DM was established by documented ICD diagnostic codes and use of anti-diabetic agents so as to match the follow-up nature of guideline diagnosis. Although there was risk that we might have excluded some patients presenting with only mild hyperglycemia after transplantation, the rationale behind the way we defined PTDM can be justified by one study showing that being diagnosed with PTDM may be of unique prognostic value rather than just having hyperglycemia [41]. However, the “heightened threshold” of diagnosis in our study might help ensure the accuracy that all the patients captured were undoubtedly diabetic and be more adaptive to the clinical setting when a new diagnosis of DM is made.

## Conclusions

To the best of our knowledge, this study is the first to provide Taiwanese population-based information about the differences in graft and patient survival outcomes among kidney transplant recipients who were stratified by DM status. In a large cohort of patients from the nationwide registry data, we found that the incidence of PTDM peaked within the first year post kidney transplantation. PTDM was associated with worse patient and graft outcomes, with the magnitude of cardiovascular and survival disadvantages from PTDM more pronounced in recipients aged less than 55 years. Although the reasons for these associations have not been disclosed, the results of our research could be a useful aid for decision makers in establishing a personalized surveillance and treatment programs.

## Supplementary information

**Additional file 1.** Additional figure and tables.

## Data Availability

All data analyzed during this study are included in this article.
