# Supplementary material for: Temporal trends of incident diabetes mellitus and subsequent outcomes in patients receiving kidney transplantation: a national cohort study in Taiwan
Source: Diabetol Metab Syndr. 2020 Apr 28;12:34. doi: 10.1186/s13098-020-00541-3 (PMC7189729; doi:10.1186/s13098-020-00541-3)
Supplement: Supplementary file 1 — Additional file 1. Additional figure and tables. [file 13098_2020_541_MOESM1_ESM.docx]

Additional file 1: Figure S1. Study design


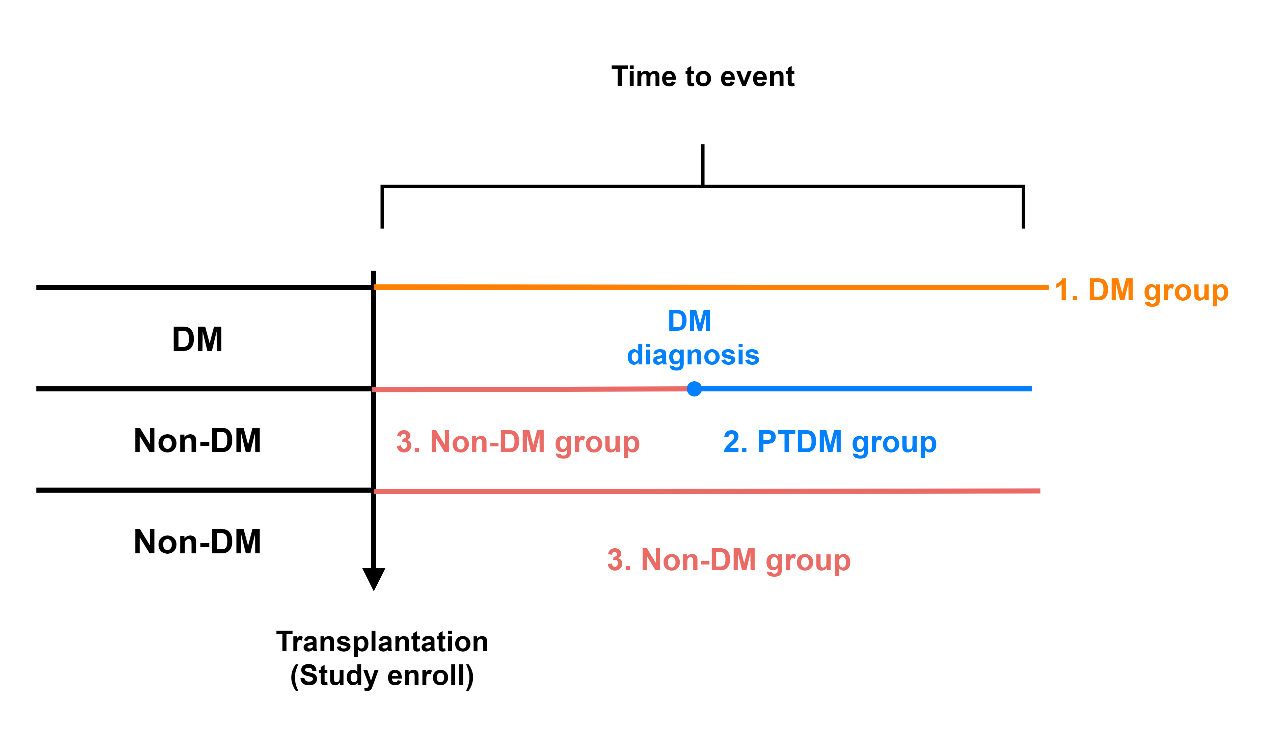


Abbreviations: DM, diabetes mellitus; PTDM, post-transplant diabetes mellitus; IQR, interquartile range.

*The median (IQR) of observation interval was 7.7 (4.5-10.6) years for overall study population from study enrollment to death, or the end of ~~the~~ follow-up period (December 31, 2013), whichever came first.

Additional file 1: Table S1. Risk of PTDM among 3,132 patients without DM prior to transplantation.

| Variables | Cox model | |  | Cox model with  competing risk | |
| --- | --- | --- | --- | --- | --- |
|  | HR (95% CI) ^a^ | p-value ^b^ |  | HR (95% CI) ^a^ | p-value ^b^ |
| Age (Unit: year) | 1.03 (1.03-1.04) | <.001* |  | 1.03 (1.02-1.04) | <.001* |
| Male (REF: Female) | 1.21 (1.03-1.43) | 0.020* |  | 1.20 (1.01-1.42) | 0.040* |
| Comorbidities |  |  |  |  |  |
| Malignancy | 1.64 (1.02-2.63) | 0.041* |  | 1.65 (1.00-2.72) | 0.048* |
| Hypertension | 1.02 (0.87-1.20) | 0.810 |  | 1.02 (0.86-1.22) | 0.806 |
| Hyperlipidemia | 1.43 (1.17-1.75) | <.001* |  | 1.44 (1.18-1.77) | <.001* |
| Cerebrovascular disease (including stroke) | 1.09 (0.72-1.64) | 0.685 |  | 1.05 (0.68-1.62) | 0.842 |
| Myocardial infarction | 2.14 (0.93-4.94) | 0.074 |  | 1.80 (0.77-4.21) | 0.176 |
| Congestive heart failure | 1.21 (0.89-1.64) | 0.231 |  | 1.14 (0.83-1.58) | 0.424 |
| Peripheral vascular disease | 1.21 (0.81-1.81) | 0.349 |  | 1.06 (0.68-1.65) | 0.806 |
| Atrial fibrillation | 1.42 (0.63-3.21) | 0.400 |  | 0.85 (0.29-2.45) | 0.759 |
| COPD | 1.32 (0.94-1.83) | 0.105 |  | 1.36 (0.98-1.89) | 0.070 |
| Liver cirrhosis | 1.27 (0.78-2.07) | 0.344 |  | 1.18 (0.62-2.24) | 0.616 |
| HBV | 1.22 (0.30-4.93) | 0.782 |  | 1.09 (0.33-3.65) | 0.886 |
| HCV | 3.25 (0.45-23.54) | 0.244 |  | 1.20 (0.11-12.86) | 0.880 |

Abbreviations: PTDM, post-transplant diabetes mellitus; DM, diabetes mellitus; HR, hazard ratio; CI, confidence interval; REF, reference; CCI, Charlson comorbidity index; COPD, chronic obstructive pulmonary disease; HBV, hepatitis B virus; HCV, hepatitis C virus.

^a^ HR and 95% CI were obtained from the Cox proportional hazard model adjusted for sex, age, comorbidity scores, place of residence, income levels, occupations, comorbidities, and medications listed in the table 1.

^b^ * means the p-value reached the statistical significance

Additional file 1: Table S2. Risk of graft failure, MACE, and all-cause mortality in kidney transplant recipients stratified by diabetes status in a detailed manner.

| DM  Status* | Graft failure | | | |  | MACE | | | |  | All-cause mortality | |
| --- | --- | --- | --- | --- | --- | --- | --- | --- | --- | --- | --- | --- |
|  | Cox model | | Cox model and  competing risk | |  | Cox model | | Cox model and  competing risk | |  | Cox model | |
|  | HR (95% CI) | P | HR (95% CI) | p |  | HR (95% CI) | p | HR (95% CI) | p |  | HR (95% CI) | p |
| Non-DM | (Reference) |  | (Reference) |  |  | (Reference) |  | (Reference) |  |  | (Reference) |  |
| PTDM | 1.75 (1.56-1.96) | <.001 | 1.65 (1.47-1.85) | <.001 |  | 1.59 (1.38-1.84) | <.001 | 1.51 (1.31-1.74) | <.001 |  | 1.79 (1.59-2.01) | <.001 |
| Duration <3 years | 1.84 (1.63-2.08) | <.001 | 1.71 (1.52-1.93) | <.001 |  | 1.60 (1.39-1.86) | <.001 | 1.49 (1.29-1.73) | <.001 |  | 1.77 (1.56-2.00) | <.001 |
| Duration 3 or more years | 1.46 (1.20-1.77) | <.001 | 1.42 (1.17-1.72) | <.001 |  | 1.34 (1.05-1.70) | 0.017 | 1.33 (1.05-1.69) | 0.018 |  | 1.87 (1.56-2.23) | <.001 |
| DM | 1.40 (1.24-1.57) | <.001 | 1.33 (1.18-1.50) | <.001 |  | 1.74 (1.50-2.02) | <.001 | 1.64 (1.41-1.90) | <.001 |  | 2.03 (1.81-2.28) | <.001 |
| Only Type 2 | 1.33 (1.18-1.51) | <.001 | 1.25 (1.10-1.42) | <.001 |  | 1.85 (1.59-2.15) | <.001 | 1.71 (1.47-1.99) | <.001 |  | 2.23 (1.99-2.51) | <.001 |

Abbreviations: MACE, major adverse cardiovascular events; HR, hazard ratio; CI, confidence interval; DM, diabetes mellitus; PTDM, post-transplant diabetes mellitus.

*All HRs (95% CI) were calculated by using Cox proportional hazards model with counting process accounted for time-dependent variables and weighted by the propensity scores. Propensity scores for the five groups stratified by DM status were calculated from variables of gender, age, Charlson comorbidity scores, place of residence, income levels, occupations, presence of comorbidities (including malignancy, hypertension, hyperlipidemia, cerebrovascular disease, myocardial infarction, congestive heart failure, peripheral vascular disease, atrial fibrillation, chronic obstructive pulmonary disease, liver cirrhosis, hepatitis B virus, hepatitis C virus), cyclosporin, tacrolimus, mycophenolate mofetil, mammalian target of rapamycin inhibitor, steroid, kidney transplantation rejection and cytomegalovirus infection. Competing risk of death with functioning graft was calculated as informative death-censoring mechanism for evaluating the outcome model of graft failure excluding death with functioning graft. Competing risk of death without experiencing MACE was calculated as informative death-censoring mechanism for evaluating the outcome model of MACE.
